# A lymphatic dwelling filarioid nematode, *Rumenfilaria andersoni* (Filarioidea; Splendidofilariinae), is an emerging parasite in Finnish cervids

**DOI:** 10.1186/s13071-015-0835-0

**Published:** 2015-04-16

**Authors:** Sauli Laaksonen, Antti Oksanen, Eric Hoberg

**Affiliations:** Finnish Food Safety Authority (Evira), Mustialankatu 3, FI-00790 Helsinki, Finland; Finnish Food Safety Authority Evira (FINPAR), Elektroniikkatie 3, FI-90590 Oulu, Finland; US National Parasite Collection, ARS, USDA, Animal Parasitic Diseases Laboratory, BARC East 1180, 10300 Baltimore Avenue, Beltsville, MD 20705 USA

**Keywords:** Lymphatic filariosis, *Rumenfilaria andersoni*, Cervids, Emerging parasites, Climate change

## Abstract

**Background:**

Recent studies revealed expansion of filarioid nematodes into northern Finland. In addition to *Setaria tundra*, an abundant filarioid, *Rumenfilaria andersoni*, was found inhabiting the lymphatic vessels of reindeer. Our study explores the dynamics of the rapid geographic expansion of *R. andersoni*, defining prevalence and density of microfilariae among 4 new cervid host species in Finland while developing a context for host-parasite ecology in Fennoscandia and more broadly in the Arctic and boreal regions.

**Methods:**

Blood samples were evaluated for presence of microfilariae from 1576 semi-domesticated reindeer, 8 captive reindeer, and free-ranging cervids including 105 wild forest reindeer, 862 moose, 114 white tailed deer and 73 roe deer in 2003–2006 (−2010). Additionally, the prepatent period and the efficacy of ivermectin treatment were investigated.

**Results:**

*Rumenfilaria andersoni* was found to be a common and abundant parasite in reindeer (0-90%) and wild forest reindeer (41-100%). Also moose (0-12%), white-tailed deer (15-22%) and roe deer (3%) were revealed as definitive hosts. Ivermectin was not effective against adult parasites. The prepatent period was estimated to be about five months.

**Conclusions:**

*Rumenfilaria andersoni* was identified in 3 endemic cervid species and the introduced white-tailed deer, all constituting previously unrecognized host species in the Palearctic. Among moose, the prevalence and intensity were substantially lower than levels observed among subspecies of reindeer. White-tailed deer had a relatively high prevalence and density of *R. andersoni* microfilariae (rmf), whereas our limited data for roe deer indicated that the nematode may not have been abundant. Density and prevalence of rmf in moose and white tailed deer suggests the nematode may be adapted to these species, and that these cervids may be among the primary hosts of *R. andersoni* and reservoirs for transmission in Finland. Our current data suggest that *R. andersoni* became established in Finland recently, coincidental with introduction of white-tailed deer from North America in 1935; subsequent invasion and emergence in the past 70–80 years appears driven by climate-related factors. An alternative hypothesis for a temporally deeper occurrence for *R. andersoni* in Fennoscandia, representing post-Pleistocene range expansion with moose tracking deglaciation, is not firmly supported.

## Background

Worldwide, filarioses represent major health hazards with important medical, veterinary and economic implications [1]. There is recent evidence documenting the range expansion of filarioid parasites of free-ranging ungulates to subarctic areas including Finland, along with an array of diseases associated with these nematode pathogens [2,3]. At northern latitudes species of several filarioid genera are known circulating among ungulate definitive hosts and various hematophagous insects as vectors [2]. Each adult female filarioid worm produces thousands of larval stages, microfilariae (mf) daily [4]; for example, *Setaria labiatopapillosa* contains at least 50,000 [4] and *Setaria tundra* over 200,000 mf [5] in the uterus. Microfilariae occur in the circulatory system or in the skin of an ungulate host where they are available to arthropod intermediate hosts (vectors) during blood meals; in the latter host, the microfilaria exsheathes, penetrates the gut wall, migrates to the haemocoel and develops to an infective stage. Vectors of different filarioid nematodes include most of the major arthropod groups known to feed on the blood of higher vertebrates, i.e. biting midges, blackflies, horse and deer flies, mosquitoes, lice, fleas, mites and ticks [6].

Microfilariae can be demonstrated in definitive hosts from blood (or skin) samples and are thus a good means for establishing diagnostics of infection among free-ranging and domestic ungulates [6]. Occurrence of *Setaria* sp. mf has previously been reported in reindeer (*Rangifer tarandus tarandus*) blood from Alaska [7], and from Sweden [8]. In Finland these parasites were demonstrated among semi-domesticated reindeer and wild cervids during the last decade [3]. Notably, a maximum of over 4000 *S. tundra* mf/ml was found in samples of sub-adult reindeer blood during the seasonally defined peak period of microfilaraemia extending from the early June until mid-September [3]. Studies in Fennoscandia, apart from Finland, have not demonstrated the presence of mf from other filarioids in the blood circulation among cervids.

In 2003 an outbreak of parasitic peritonitis, caused by *S. tundra*, emerged in the Finnish reindeer population [2,3]. This outbreak was the third to have been documented, following events in 1973 among reindeer and 1989 among moose (*Alces alces*) [2,3]. The outbreaks were associated with warm summer weather conditions, specifically mean temperatures during the summer months exceeding 14°C [9].

**Figure 1:**
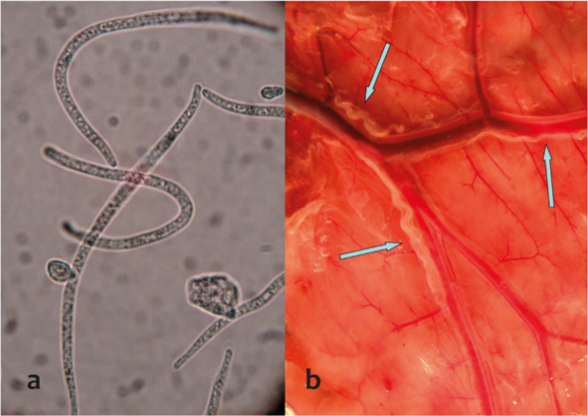
*Rumenfilaria andersoni* microfilriae **(a)** in cervids blood circulation and an adult parasite **(b)** in the lymphatic vessels adjacent to the rumen of reindeer.

Epidemiological studies of mf of *S. tundra* in reindeer blood [3] led to the discovery of a new/unidentified filarioid in January 2004. These mf (Figure 1a) were abundant in blood samples from cervids, especially reindeer. Subsequently, adult parasites (Figure 1b) were found inhabiting the lymphatic vessels adjacent to the rumen of adult hosts, based on necropsies performed in December 2006. Identity of these previously unrecognized filarioids was shown, based on comparative morphology, to be *Rumenfilaria andersoni* Lankester and Snider, 1982 (Splendidofilariinae), a species considered to be endemic to North America and which had not been documented in Eurasian hosts and localities [10]. This nematode was originally described in one moose (*Alces americanus*) from eastern Canada. The authors could not accurately describe the localization in the host but considered that adult nematodes were in the venous system [11]. In North America, for many years, the parasite was known only from the original description. *Rumenfilaria* was recently documented in Alaska where it occurs in over 70% of moose (based on mf), suggesting a broad geographic range in the Nearctic [12]. Interestingly, it has not been reported in subspecies of caribou or other high latitude ungulates, nor in other cervids including species of *Cervus* and *Odocoileus* in the temperate and boreal zone of North America, nor is it apparently known from other localities across Eurasia [10,12]. The detection of adult *R. andersoni* nematodes in reindeer represents the first lymphatic filarioid to be recognized in artiodactyles, which make our findings especially significant.

The main objective of our study was to monitor the dynamics of *R. andersoni* during a period of an apparently rapid geographic range expansion in Finnish Lapland. To achieve this, we explored basic biology of the parasite, including life history parameters such as host distribution, infection reservoirs, prepatent period, life span of adult nematodes and duration of microfilaraemia. One of the aims was also to collect an extensive data set about the prevalence of filarioid nematodes in cervids during an outbreak, which would serve as a reliable baseline for future monitoring. To achieve these goals, we collected and archived parasitological samples from reindeer and other cervid species over a large spatial and temporal scale. These data contributed to a developing understanding of the impact of this apparently new parasite for northern boreal ecosystems of Fennoscandia and also provide insights for faunal dynamics across the Holarctic.

## Methods

### Sampling of hosts and localities

Sampling was designed to explore the distribution of filarioid nematodes based on detection of microfilariae in blood samples acquired from reindeer and other potential cervid hosts in Finland and in some cases more broadly in Fennoscandia. Our sampling protocols included semi-domesticated reindeer, captive reindeer, and other wild, free-ranging cervids as outlined below.

#### Microfilariae detection

The blood samples were collected during exsanguination after stunning or shooting (wild cervids); the blood flow from cut jugular arteries and veins was directed into opened blood tubes (Venosafe EDTA, Terumo, Belgium). The samples (1 ml blood/animal) were examined for the presence of *Rumenfilaria* mf (rmf) by the modified Knott’s technique [13] and the rmf were counted in temporary wet mounts to ten thousands. The presence and identity of mf in cervid blood circulation, was based on comparative morphological characters [10].

#### Statistical analyses

**Figure 2:**
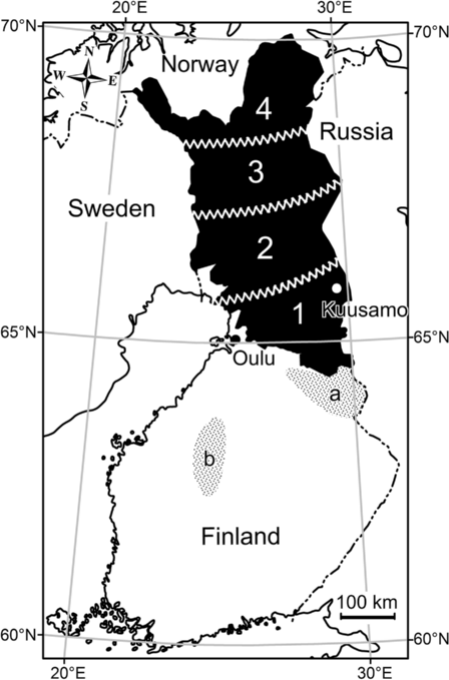
Finnish reindeer herding area (black) divided into four sub areas for analysis of *R. adersoni* nicrofilariae (rmf) in cervids. Dotted areas mark the northern (a; Kainuu area) and the southern (b; Suomenselkä area) populations of wild forest reindeer.

Statistical analyses were performed with Stata 9 (StataCorp LP, USA) software. The reindeer husbandry area was divided in four subareas (Figure 2) for analyses of spatial emergence of these parasites. In modeling, sub-area was used as a hierarchical dummy variable, and as a result, differences between adjacent sub-areas were evaluated and compared. Overall effect of the age group and year on the prevalence and density of rmf in blood was analyzed using logistic regression and Poisson model, respectively. Rmf counts in blood were divided into 5 groups for Poisson models (0, 1–100, 101–1000, 1001–10000 and > 10000 rmf/ml). Vena/skin analyses of captive reindeer (see below) were made by using a paired t-test. Analyses of prevalence among wild cervids were conducted using Pearson’s chi square test and in wild forest reindeer (*Rangifer tarandus fennicus*), density was compared by the Poisson model as in reindeer. The level of significance was set at 5% (p < 0.05).

#### Blood from semi-domesticated reindeer at slaughter

The total summer population of semi-domesticated reindeer in Finland during the time-frame of the study was about 300,000 individuals of which about 100,000 were slaughtered annually [14]. To analyze the spatial and temporal variation in prevalence and density of rmf and variation between adults and calves in the reindeer population, a total of 1118 blood samples (494 adults and 624 calves), selected randomly from all the regions of the Finnish reindeer herding area (Figure 2) were collected. In the winter of 2003–2004, 627 animals were sampled (273 adults and 354 calves; 20 January to 3 April) and in the winter of 2005–2006, 491 animals (221 adults and 270 calves; 1 January to 1 February).

To compare the current prevalence of the infection to that of the previous decade, 242 archived blood samples collected randomly in 1997 from throughout the reindeer herding area, were included in the study. These additional samples had been stored frozen (−20°C without anticoagulant) and were examined as described [3]. Further blood samples were collected from reindeer in one herd located in Kuusamo between 18 December 2005 through 30 January 2006 from 26 adults and 34 calves and 17 January through 1 February 2009 from 64 adults and 152 calves to allow direct comparisons with the “baseline” established in 1997 and to determine possible changes in distribution and prevalence.

The potential for infection by a transplacental pathway was also evaluated. In this regard, blood samples (single 10 ml) from 90 unborn fetuses (5^th^ – 6^th^ month gestation), near the end of the 7–8 month term of prenatal development, were collected at the Kuusamo slaughter facility in February 2004.

#### Monitoring of captive reindeer

**Figure 3:**
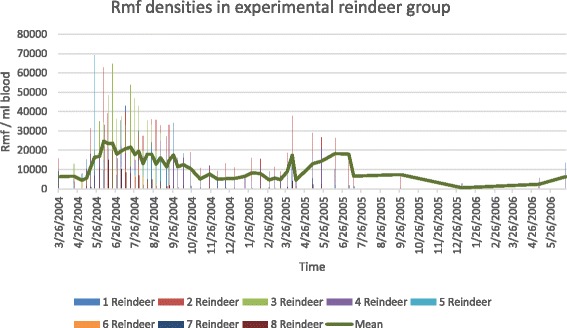
The periodicity of *R. andersoni* microfilaremia in the blood of the group of eight naturally *R. andersoni* infected reindeer in experimental zoo of Oulu.

In March 2004, three male and four female semi-domesticated reindeer calves (10 months old) and one 3-year-old female reindeer were relocated from Kuusamo to the experimental zoo of the University of Oulu (Figure 3). All reindeer were naturally infected and had rmf in circulatory blood (as well as *S. tundra* mf, see [3]). The density of mf was monitored weekly over one year by jugular vein samples collected in evacuated blood collection tubes (Venoject VP-100SDK, with 18 G Venoject needles, Terumo Corporation, Belgium). The samples were collected at 10–11:00 a.m. (see [3]).

To evaluate the circadian rhythm of the microfilaraemia, blood samples were collected for rmf monitoring every 3 h for 24 h on 18 July 2006. To evaluate the localization and density of rmf in peripheral blood circulation, samples were taken simultaeneously from the jugular vein (blood collection tubes) and from capillary vessels (haematocrit tubes) at 2–3 mm deep from the skin of the ear from 3 reindeer; sampling was 5 times during the study.

#### Blood from wild cervids

To study the possible hosts and reservoirs of *R. andersoni*, 452 blood samples were collected from wild cervids during the period from 26 January 2004 to 4 June 2005, using similar methods as described. Samples were collected by hunters. A total of 212 of these blood samples were from moose in the middle and southern part of the reindeer herding area and 112 outside the reindeer herding area. A further 92 samples were from wild forest reindeer of which 33 represented the population living just on the southern border of the reindeer herding area (Kainuu population) and 59 the population in the middle of Finland (Suomenselkä population) with no contacts with semi-domesticated reindeer (Figure 2). Other samples from adult cervids during initial surveys included 17 from roe deer (*Capreolus capreolus*) and 9 from white tailed deer (*Odocoileus virginianus*).

Subsequently, additional sampling from mainland localities resulted in blood from 415 moose in 2007 and from 78 moose during 25 November 2008 to 6 March 2009. Concurrently, samples were collected from 30 adult moose on Åland, an archipelago about 150 km from the southwestern main land. Elsewhere, on the mainland sampling included 105 white-tailed deer (4 January 2007 to 7 February 2008), 56 roe deer (6 October to 31 December, and 13 wild forest reindeer (9 November 2007 to 2 April 2008). In 2010 samples were collected from 15 adult moose in central and northern Sweden.

The summer population of Finnish moose during the study period was about 90,000 animals. Roe deer were less abundant, numbering about 18,000, and also widely distributed across Finland. White-tailed deer, introduced in 1935 from the USA, had an estimated population size of about 48,000 animals, with a range localized mostly to southwestern Finland, where the samples were collected for this study. A total of 2000 individuals of Finnish wild forest reindeer were divided equally into northern (Kainuu) and southern (Suomenselkä) populations (Figure 2) [15].

#### Prepatent period

Estimation of the prepatent period of *R. andersoni* was derived from blood samples collected at the Kuusamo reindeer slaughterhouse (Figure 2) from 145 adults and 240 calves. There were 8 slaughter-batches examined from October 2004 to January 2005. All slaughter batches originated from the same pastures in Kuusamo. The calves were assumed to have been infected during the summer swarming period of blood-feeding insects.

#### Antiparasitic treatment trial

The efficacy of antiparasitic treatment in controlling infections was also investigated as an extension of epidemiological studies. Studies involved 21 adult reindeer from the Kaamanen reindeer research station in Inari, northern Lapland. They received ivermectin injection on 29 November 2006, (Bimectin ®, Vetpharma AB) s.c. in the neck (200 μg/kg b.w). In the beginning of the trial all reindeer were rmf positive, mean 219 rmf/ml blood (range 1–1000). Blood samples were collected from the vena jugularis (Venoject® VP-100SDK) with 12G Venoject® needles. Subsequently, detection, recognition, identification and counting of mf were performed as previously described [3]. Control samples for rmf detection were taken on 11 December 2006, 10 January 2007, 6 February 2007, 5 March 2007 and 17 April 2007.

All the animal handling procedures for this work were approved by the Experimental Animal Committee, the University of Oulu (license no. 030/04).

## Results

During 2004–2006, *R. andersoni* was found to be a common and abundant parasite in reindeer. We documented the distribution of the parasite in adults and calves of multiple reindeer herds, and demonstrated the presence of the parasite in all species of cervids, sampled in Finland. Based on comparison to samples from 1997, there has been an apparent broadening or expanding distribution for this filarioid in Finland consistent with previous introduction and invasion. We examine specific attributes of these data and infections.

### Sampling of hosts and localities

#### Semi-domesticated reindeer at slaughter

Overall rmf occurred in 64% (718 of 1118) of reindeer blood samples, with a mean density of 452 rmf/ml blood (range 1–19400, SD 1454). The prevalence (376/494, 76%) (p < 0.001) and density (mean 678 rmf/ml, range 1–19400, SD1905) (p < 0.001) of rmf was higher in adults than in calves (55% (342/624); mean density 204, range 1–7300 rmf/ml, SD 581). Among both age classes of hosts, infection intensity decreased from the south to the north with the parasite apparently emergent in northernmost Finland (Table 1).

**Table 1:** **The spatial** ***R. andersoni*** **microfilariae (rmf) prevalence in semi-domesticated reindeer in 2004 and 2006 in four collection areas**

**Area (south to north) Figure** 2	**Year**	**Number of reindeer**		**Rmf prevalence**	**Diff. between years chi p**	**Mixed infect with** ***Setaria tundra***
1	2004	calf	116	94%	0.000	95%
	2006	calf	114	45%		33%
	2004	adult	133	96%	0.001	49%
	2006	adult	87	83%		17%
2	2004	calf	150	90%	0.000	80%
	2006	calf	56	43%		15%
	2004	adult	82	87%	0.438	36%
	2006	adult	37	81%		17%
3	2004	calf	31	81%	0.000	19%
	2006	calf	25	8%		8%
	2004	adult	17	88%	0.208	17%
	2006	adult	25	72%		40%
4	2004	calf	57	0%	0.214	0%
	2006	calf	75	3%		0%
	2004	adult	41	0%	0.000	0%
	2006	adult	72	42%		2%

Data collected from slaughter reindeer.

In 1997, rmf were present at very low density (mean 8.5 rmf/ml, range 1–35) in 8% of samples (19/242). All infected reindeer were from the southern part of the Finnish reindeer herding area (subarea 1, Figure 2).

In the Kuusamo area (Figure 2) during 18 December 2005–30 January 2006, 92% of the adults were rmf positive, mean 1973 rmf/ml blood (SD 4–19400) and 18% of the calves, mean 60 (range 1 – 23). During 1–17 February 2009, 56% of the adults were rmf positive (mean 256 rmf/ml, range 1–1363) but all 152 calves were rmf negative.

The occurrence of transplacental infection was not demonstrated. Microfilariae of *R. andersoni* or other filarioids were not found in blood samples taken from reindeer foetuses examined from the slaughterhouse.

#### Monitoring of captive reindeer

In the experimental reindeer group, the peak period of microfilaraemia was from early May to the end of September 2004, with the mean number of 16923 rmf/ml blood (range 3810–35321) (Figure 3). During the peak microfilaraemia, in three individual reindeer the rmf density was over 60000 rmf/ml blood. After that, the density decreased and remained at a lower level (mean 7193, range 34–16000); subsequently after early April in the following year a secondary peak was observed during the summer, although lower in intensity than the first summer peak (14 June 2005, mean 18300 rmf/ml, range 9–24600). After the secondary peak, the rmf concentration was persistent but remained low over winter, rising again in the third summer in 2006, to the mean level of 6391 rmf/ml (range 131–13431) on 20 July and reindeer were still harbouring circulating rmf in autumn 2006 (Figure 3). In the summer of 2004 one reindeer calf was imported to the zoo from North Lapland, supposedly free of infection, and later it developed a substantial rmf infection.

**Figure 4:**
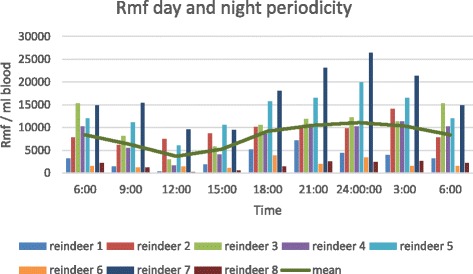
The circadian rhythm of the *Rumenfilaria andersoni* microfilaraemia in the group of eight naturally infected reindeer in experimental zoo of Oulu, 18 July 2006.

**Figure 5:**
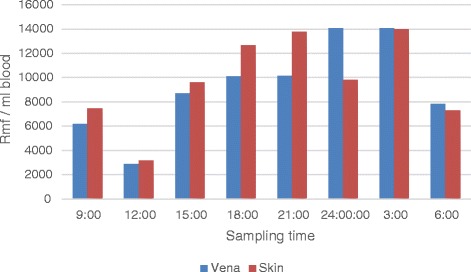
Comparison of *Rumenfilaria andersoni* microfilaria (rmf) counts in vena and skin samples in three naturally infected reindeer in experimental zoo of Oulu, 18 July 2006. x-axis; time of sampling pairs, y-axis; mean rmf/ml blood.

In the round-the-clock monitoring, peak microfilaraemia, over 10000 rmf/ml, occurred at night between 21:00 and 03:00, with the maximum number of microfilariae in the blood (13500 /ml) at midnight (mean 11089 rmf/ml). After that the density of rmf gradually decreased to the minimum of 3690 rmf/ml at 12 noon (Figure 4). There were no differences between microfilarial densities sampled from the jugular vena and from capillary circulation (p = 0.703) (Figure 5).

The entire experimental reindeer group revealed decreased general condition during the summer of 2004 when the mf values (both *S. tundra* [3] and *R. andersoni*) were at the peak; in the one imported reindeer, body condition was poor coincidental with the peak for mf in 2005. Body condition was low, mainly 1 (scale 1 to 4) [2,3] and the fur was dry and tangled and there was unexplained itch, stiffness and lameness. An improvement in apparent body condition was observed during the summer 2005 when the mf values were lower.

#### Wild cervids

The results of rmf monitoring in wild cervids are presented in Table 2.

**Table 2:** ***Rumenfilaria andersoni*** **(rmf) microfilariae detected in the blood of over eight month old wild cervids between 26 January 2004 and 4 June 2005 in Finland**

**Species/area**	**No of animals**	**Rmf prevalence**		**Mean density (range)**	
**Moose, outside reindeer herding area**	112	8%		127 (0–574)	
**Moose, in reindeer herding area**	212	7.5%		92 (1–533)	
**Wild forest reindeer, Kainuu**	33	48%	p < 0.04	3752 (0–48000)	p < 0.03
**Wild forest reindeer, Suomenselkä**	59	71%		596 (0–12600)	
**Roe deer**	17	0		0	
**White-tailed deer**	9	22%		446 (3–890)	

Density as number of *R. andersoni* microfilariae/ml.

Surveys conducted during 2004 and 2005 indicated that there were no differences between the prevalence of rmf (7.5%) in moose from the reindeer herding area and adjacent habitats with no reindeer (8%). In wild forest reindeer *R. andersoni* infection was more prevalent (p < 0.039) in the Suomenselkä region (71%) than in Kainuu, just on the border of the reindeer herding area (41%). The intensity of infection was heavier (p < 0.026) in Kainuu (mean 3752 rmf/ml, range 1–48000) than in Suomenselkä (mean 596 rmf/ml, range 1–12600). No rmf were detected in roe deer during initial survey. Of nine white tailed deer, 22% harbored rmf infection (Table 3).

**Table 3:** **The prevalences and densities (rmf/ml blood) of** ***R. andersoni*** **microfilaremia in reindeer blood in eight slaughter batches at Kuusamo slaughterhouse in October 2004 to January 2005**

**Slaughter day**	**30.10.04**	**4.11.04**	**11.11.04**	**18.11.04**	**30.11.04**	**15.12.04**	**5.1.05**	**24.1.05**
**Calves prevalence/density**	34	22	28	41	30	18	27	40
	0/0	0/0	0/0	0/0	0/0	0/0	4/10	18/60
**Adults prevalence/density**			21	39	27	10	24	24
			95/1259	87/2263	93/994	100/498	67/1973	100/4227

Subsequent sampling during 2007, 2008 and 2009 demonstrated the presence of *R. andersoni* in all species of Finnish cervids examined. In 2007 of 415 animals from all over Finland, 48 (12%) harbored rmf. During 2008 and 2009 among 78 moose from the mainland 6 (8%) were rmf positive with a mean density of 308 rmf/ml (range, 3–1154). All moose sampled from Åland (in the absence of white tailed deer) were negative for rmf. Among 105 white-tailed deer 16 (15%) were positive, with a mean of 10 rmf/ml (range 2–16); among 59 roe deer 2 (3%) were positive, with 2–6 rmf/ml; and among 13 wild forest reindeer from the Kainuu population, all (100%) were positive with a mean of 1048 rmf/ml (range 13–6172). Also, based on samples from 2010, only one of 15 Swedish moose, near the Finnish border in Piteå, was positive, having two rmf/ml blood.

#### Prepatent period

In the study to determine the prepatent period, calves were shown to be free of rmf in the beginning of the slaughter season during November, whereas 95% of adult reindeer were positive. Rmf emerged in blood circulation among calves in the beginning of January, indicating an approximately 5 month period of development, or more, assuming infection after the peak microfilaraemia (Figure 3) and allowing time for development in the arthropod vector. Among adult reindeer, infections appeared to be persistent over time, with prevalence of rmf remaining between 80 to 100% through the slaughter season (Table 3).

#### Anthelmintic treatment

The rmf values observed in circulating blood after ivermectin treatment varied with date of sampling. Post-treatment samples demonstrated a period of recovery following drug administration as shown by prevalence and intensity (density of rmf) of infection (treatment 29 November): 11 December 2006, prevalence 29%, mean 4 rmf/ ml (range 1–11); 10 January 2007, prevalence 0%; 6 February 2007, prevalence 38%, mean 5 rmf/ml, (range 1–13); 5 March 2007, prevalence 52%, mean 20 rmf/ml (range 5 – 53); and 17 April 2007, prevalence 71%, mean 52 rmf/ml (range 2–195) respectively. Data for infections in the control group are those outlined previously, including prevalence and counts from slaughtered or captive reindeer. Irrespective of study group, the density of rmf was observed to increase from winter into spring and summer (Tables 1 and 3, Figure 3).

## Discussion

### Discovery of *R. andersoni* in Finland

The occurrence of *R. andersoni* in cervid hosts from Fennoscandia was unknown prior to 2004 when microfilariae of these nematodes were first discovered in survey for the related *S. tundra* during an outbreak of parasitic peritonitis among semi-domesticated reindeer [2,3]. The specific identity of the mf was a mystery until adult nematodes were discovered. The adult nematodes from the lymphatic system, adjacent to the rumen, were later collected and identified, based on morphological criteria [10], in reindeer and moose during 2006.

Subsequent broad-based surveys in Finland to establish geographic distribution and host range between 2004 and 2009 assessed the prevalence and intensity of rmf in blood samples from the circulatory system of cervids. In addition to semi-domesticated reindeer, *R. andersoni* was identified in 3 endemic cervids (moose, wild forest reindeer and roe deer) and the exotic or introduced white-tailed deer, all constituting previously unrecognized host species in Fennoscandia.

Prior to discovery and collection of the adults, microfilariae of *R. andersoni* were recognized [10] to belong to the superfamily Filarioidea (Spirurida), which all are transmitted by haematophagous arthropods [6]. The prevalence and density of rmf in reindeer blood was extremely high (Table 1). Based on the occurrence of rmf (Table 1), infection was more prevalent among semi- domesticated reindeer from the southern areas than from the north, indicating a geographic and temporal gradient in the distribution of infections. In contrast, *S. tundra* infections did not show this pronounced gradient between southern and northern populations of wild forest reindeer; infections of *S. tundra* were more common in the Kainuu population with contacts to semi-domesticated reindeer [3]. Among moose, the risk of infection by *R. andersoni* was independent of reindeer herding, and the overall prevalence in these large cervids was substantially lower than levels observed among subspecies of reindeer. White-tailed deer had a relatively high prevalence and density of rmf, whereas our limited data for roe deer indicated that the nematodes may not be abundant (Table 2).

The density and prevalence of rmf in moose and white-tailed deer suggests the nematode may be adapted to these species, and that these cervids may be among the primary hosts of *R. andersoni* and reservoirs of rmf in Finland and perhaps elsewhere. In contrast, elevated prevalence and density in reindeer may reflect absence of an effective immunity against these nematodes in an otherwise naive host species. Another possible explanation might be that transmission to reindeer is more efficient, although there are no specific data currently to evaluate this point. Overall, distributions appear compatible with host colonization under changing patterns of geographic expansion. [16-18].

### A history of distribution for *R. andersoni* in Finland

Filarioids including species of *Setaria* and *Onchocerca* occur across the Holarctic among artiodactyl hosts, and although the Palearctic fauna includes additional genera and species, lymphatic dwelling filarioids had been unknown among ungulates of the world, including Eurasian cervids except those reported in Finland [10,19-21]. Studies in Fennoscandia had demonstrated *S. tundra* among cervids, but the presence of circulatory mf of other genera and species of filarioids had not been observed until the apparent emergence of *R. andersoni* in Finland [10]. Until recently, knowledge about the distribution of this filarioid was limited to the original description based on nematodes in a moose collected from Ontario, Canada [11] and to description in Finnish reindeer [10]. The parasite is now assumed to occur across most of the northern Nearctic, following documentation of abundant infections in moose from Alaska [12], but there are no additional records in any cervids from the Palearctic. Limited sampling for these otherwise cryptic nematodes in North America, however, is likely to have biased our understanding of the true distribution geographically among other cervids, including deer and caribou [12]. Filarioid faunal diversity in the Palearctic has received attention, as outlined above, but survey and inventory in eastern Eurasia or even Central Europe may have been insufficient to demonstrate the occurrence of these filarioids.

Our current understanding of filarioid diversity, host associations and biogeography, although incomplete, suggests two alternative hypotheses for the occurrence and the establishment of *R. andersoni* in Finland. Although geographic expansion in Finland and Fennoscandia appears recent and ongoing, the initial origin of this parasite population requires resolution and can be linked either to (1) post-glacial dispersal with moose and reindeer from Russia and western Europe [15], or (2) anthropogenic introduction within the past 80 years, coincidental with the translocation of white-tailed deer from North America in 1935 [22].

Geographic colonisation of parasites with moose from Central Europe via Jutland and from the east via Russia after the last glacial [23], about ten thousand years ago, appears unlikely based on our current understanding of distribution for *R. andersoni*, with no previous records from Eurasia. A more plausible mechanism is jump dispersal and focal establishment of *R. andersoni* coincidental with origins of the current population of white-tailed deer in Finland which resulted from a single introduction in 1935 [22]. The original and only translocation involved 3 male and 4 female wild fawns that were captured in Minnesota and presented as a gift to Finland by Finnish immigrants from the upper central plains of the United States. Only 1 male and 4 females survived the trans-Atlantic passage by ship to Helsinki. Introduction was successful, however, and subsequent reproduction led to the present unique population in Europe of about 48,000 deer concentrated in Southern and Western Finland.

A hypothesis for anthropogenically driven introduction with *O. virginianus* is dependent on our understanding of the distribution of *R. andersoni* in the Nearctic and its associations with cervids. Morphological uniformity of rmf in Finland and Alaska, or of adult parasites in Finland and Ontario, does not resolve the history, and exploration of genetic diversity in filarioids from geographically disjunct host populations would be required [10,12]. In this regard, sequence-based comparisons of Nearctic and Finnish populations can serve to test or refute the competing hypotheses for origin in Fennoscandia. Limited variation in molecular sequence data at several loci, without indication of diversity or divergence, would corroborate a recent introduction, and would otherwise be inconsistent with an extended history of geographic distribution and isolation encompassing the Holarctic during the Pleistocene.

Caveats aside, survey and monitoring since 2004 indicates expansion from a regionally limited or focal geographic distribution for *R. andersoni* in Finland. *Rumenfilaria andersoni* occurs among an assemblage of sympatric cervids (moose, reindeer, and less often roe deer) including introduced white-tailed deer. Although *R. andersoni* is observed within this assemblage of cervids, the parasite has also been documented in localities in the absence of white-tailed deer in Lapland and Alaska [12]. Further, differences in relative prevalence between moose in Alaska (at 70%) [12] and Finland (7-8%) may suggest a developing or emergent association in Fennoscandia, further consistent with a more recent introduction, perhaps with white tailed deer.

Over the past decade, parasites have apparently been under expansion from south to north involving geographic and host colonization. The cline or gradient in abundance and prevalence of *R. andersoni* is consistent with introduction, establishment and subsequent invasion in Finland of an otherwise exotic parasite species with its typical host [24]. As such, the ongoing events of expansion provide an example of the role of ecological fitting and sloppy fitness space within a multi-level and complex assemblage of hosts and parasites where colonization has been facilitated among arrays of new cervid hosts and new hematophagous insects required as intermediate hosts and vectors for transmission [17,18,24,25].

### Seasonal transmission and occurrence among hosts

The lifespan and patterns of larval production for adults of *R. andersoni* in the lymphatic system are unknown as is the duration of viability and circulation of rmf in the blood of definitive hosts. The discovery of high prevalence and density of rmf in adult reindeer already in autumn suggests that these represent old, well established and persistent infections, and potentially that the process of infection may be cumulative. In contrast, calves did not demonstrate rmf in circulation until the middle of December, indicative of a relatively long prepatent period (perhaps over 5 months) and infections established from events of blood feeding in the previous summer by haematophagous vectors. In experimental reindeer the rmf prevalence was persistent for at least three years, although recurrent peaks of rmf density occurred during successive summers. These recurrent peaks diminished over time in captive animals, suggesting that some immunity may build up in reindeer and other cervid hosts in natural settings.

Filarioid nematodes belong to the helminth species with the highest average lifespan in their host’s body, and a common feature in lymphatic filariosis in primates is the chronic nature of infections that persist over many years [26]. Such a life history pattern reflects the capability of filarioid nematodes to modulate host immune systems resulting in tolerance of infections in the definitive host. Extended longevity for parasites in large and highly vagile ungulates would effectively serve as an invasion- buffer facilitating maintenance and dissemination under rapidly changing regimes of climate and weather, characteristics also shared with some protostrongylid lungworms at high latitudes [24,27,28]. Such temporal and spatial persistence would provide an extended window for geographic expansion, enhancing the potential for invasion and colonization involving both potential definitive and intermediate hosts and over time [24,29].

The assumption that *R. andersoni* is transmitted by haematophagous vectors, like other filarioid nematodes [6], is supported by the seasonal midsummer peak in rmf density in reindeer, and by the well-known attacks on herds of caribou/reindeer in the summer by massive swarms of blood-feeding insects [16]. Also the fact that *R. andersoni* emerged in reindeer simultaneously with an outbreak of *S. tundra* reflects common factors and determinants of transmission for these filarioids at high latitudes [9,30]. The high prevalence and density in the blood circulation, substantial summer peaks of abundance coinciding with räkkä-time (a local term that recognizes the seasonal period of peak activity by blood feeding insects [30]), its appearance in calves in early winter after an extended prepatent period of about 5 months and apparently rapid emergence in reindeer populations reveals that the vector is effective. Interestingly, almost 1000 *Aedes* spp. mosquitoes were dissected during the study of the outbreak of *S. tundra*, but no larvae other than those of *S. tundra* were found [30]. Concurrently, in the same study in the captive reindeer, the mean counts of rmf in 2004 during the mosquito collection time were 23400 (17–18 June) and 19500 rmf/ml (3 August), indicative of considerable abundance and potential for transmission; no developing *R. andersoni* larvae were recognized in mosquitoes except some melanised unrecognizable mf. At the same time, the *S. tundra* microfilariae were considerably less abundant, with blood-counts of 1007 and 1183 mf/ml respectively; and 41% of the *Aedes* spp. mosquitoes examined harboured 1 to 51 *S. tundra* larvae [30]. These data suggest that distinctly different arrays of insect vectors may be involved in transmission for *R. andersoni* and *S. tundra*.

Microfilarial periodicity among filaroids has developed so that peak microfilaraemia coincides with the active feeding period of the insect vector. Thus, the behaviour and niches of mf have been selected under the pressure and selective regime established by feeding and activity habits of specific hematophagous insects [31]. The peak microfilaremia of *R. andersoni* during summer midnights perhaps suggests that night active mosquitoes such as species of *Culiseta* and *Anopheles* are required for development and transmission. Other potential vectors for dissemination of rmf have not been demonstrated, including potential invasive species under expansion from the south with changing conditions of climate and habitat.

### The role of climate in invasion

It is apparent that *R. andersoni* has emerged in Finnish reindeer and was undergoing northward expansion coinciding with the emergence and outbreaks of *S. tundra* [2,3,9,30]. Physical factors including temperature and humidity may influence the occurrence of both species of filarioids during seasonally defined windows for transmission [9,30]. Expansion and emergence for these filarioids may reflect changing patterns of resilience and thresholds for parasite development in differing arrays of intermediate hosts facilitated by northward shifts in the distribution of permissive environments that can support transmission [12,24,27,28]. Insect vectors can have more rapid metamorphosis with global warming and parasites may have accelerated development or incubation in mosquitoes, assuming that temperatures do not exceed tolerances and resilience that would directly influence survival and persistence. These vectors can transmit *S. tundra, R. andersoni* or other filarioid nematodes. Increasing rates of metamorphosis for mosquitoes and especially a shorter incubation of filarioid nematodes could lead to epidemics in Fennoscandia [9,30]. Thus the expansion of filarioids in Finland may to some degree parallel those processes that are driving geographic invasion and emergence of protostrongylid nematodes under the influence of climate in the Central Canadian Arctic [12,28].

### Connection to weather

Exploring a potential climate relationship for invasion and emergence, it is interesting to note the shifts in parasite abundance in relation to summer ambient temperatures (distinguishing the short-term or extreme weather events from the longer-term cumulative process of climate warming). At Kuusamo, sampling in reindeer from winter 2004 (representing transmission during the summer of 2003) demonstrated that almost 100% of adults and calves had rmf (Table 1). In the same herd in winter 2006 (transmission in summer 2005), prevalence of rmf in adults was still over 90% (reflecting persistence of infections), but in calves had decreased to under 20% (reflecting acquisition of new infections). The prevalence had already started to decrease at Kuusamo in (Table 1) 2005 simultaneously with the *S. tundra* outbreak when the focus of the *Setaria* outbreak moved to the north [2,3], and this shift appears attributable to environmental temperatures and weather during this period. Although circumstantial for *R. andersoni*, it has been demonstrated that at a mean temperature below 14°C, the development of *S. tundra* larvae is not completed in mosquitoes. It has also been demonstrated that mean summer temperatures over 14°C drive the emergence of *S. tundra* outbreaks [9]. The summer of 2008 at Kuusamo was very cold, with a mean of 11.3°C [32]. In further sampling in 2009 (transmission in summer 2008) 50% of adults still harboured rmf but not any of the 150 calves, showing that the transmission could not happen in those conditions. Thus, surveys at Kuusamo for rmf in 2006, and 2009 showed decreasing prevalence in reindeer and there was no transmission in 2008 when all calves were clear of infection.

These patterns appear to be indicative of the interplay of shifting balances in climate and weather that determine the distribution of permissive environments and conditions that facilitate establishment and maintenance of parasite populations [28]. Such intricate balances can be limiting factors of development, transmission, expansion, invasive processes and disease emergence and may define the interaction of changing zones of contact among ungulate hosts and the abundance of hematophagous vectors [9,28,30]. These interacting mechanisms are not idiosyncratic and limited to conditions in Finland, or the Central Canadian Arctic, but have relevance on a broader regional and global scale.

### Pathology and impact of *R. andersoni*

**Figure 6:**
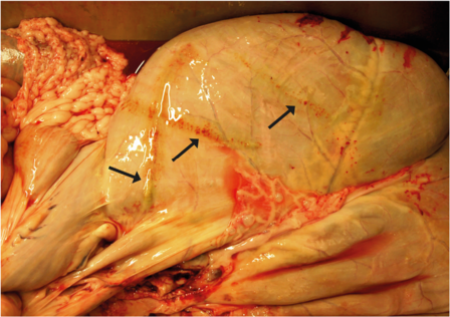
Visible greenish inflammatory changes were frequently observed around ruminal lymphatic vessel of adult reindeer inhabiting *R. andersoni* nematodes during reindeer slaughter in 2004–07.

The pathological impact of adult *R. andersoni* on reindeer health and well-being remains unknown, although macroscopic greenish inflammatory changes were frequently observed around the ruminal lymphatic vessels during reindeer slaughter in 2004–07 (S. Laaksonen, personal observations) (Figure 6). The impact of microfilaraemia on reindeer health also remains undetermined, although it is highly probable that substantial density of rmf observed in blood circulation may have negative systemic effects [33]. The eosinophilic reaction in skin and in lymph nodes reveals that high counts of rmf in blood circulation also would be predicted to have a negative impact on overall cervid health [34].

### Anthelmintic treatment and control

Endectocidic antiparasitic treatment is widely and routinely used annually in Finnish reindeer herding management when about 80% of the breeding reindeer are treated with ivermectin during late autumn round-ups or when animals are corralled during early winter [35]. Ivermectin was not efficient against adult *R. andersoni* parasites although it temporarily cleared rmf from blood circulation.

There may be host inflammatory responses resulting from microfilariae killed by the ivermectin treatment [34,36]. This might explain a period of debilitation and poor condition of animals following anthelmintic treatment as reported by local reindeer herders (S. Laaksonen, unpublished observations). To avoid these symptoms, reindeer should be treated in autumn, before *R. andersoni* parasites have reached sexual maturity and have started to produce rmf in blood circulation, parallel to recommendations in the treatment of *S. tundra* infection [35]. Post-treatment, however, *R. andersoni* microfilariae reappeared within about two months which is consistent with observations involving intervention with ivermectin to interrupt transmission of parasites in cases of human lymphatic filariosis [36]. Thus, for *R. andersoni*, chemotherapy had a transient, if any, impact on adult parasites, but was effective for a period of time in clearing microfilariae from the bloodstream and consequently could prevent the spread of parasites to mosquitoes depending on the seasonal timing of treatment. The antiparasitic treatment of reindeer is not possible during summer [35] and routine treatment takes place during late autumn or early winter with transmission window closed due to the absence of the hematophagous insects. Such a lack of complete elimination and control indicates that the ivermectin treatment as applied in management of Finnish reindeer did not prevent the movement or expansion of the *R. andersoni* outbreak to new areas in the North. This also indicates efficient transmission dynamics of the parasite.

### Consequences of expansion for *R. andersoni*

Filarioids are now abundant parasites among cervid hosts in Finland and the critical influences of climate change and what have been regarded as extremes in weather and summer temperature in accelerating geographic expansion have been clearly demonstrated [9,37]. The human-cervid interface across Fennoscandia is extensive suggesting the potential for zoonotic transmission to people. Seasonal exposure and potential infections would be mediated by the exceptional abundance and coincidence of swarming hematophagous insects, heavily infected cervid hosts harboring large populations of microfilariae, and the sympatry of reindeer, moose, white-tailed deer and people over relatively large areas. As has been noted in the context of the global fauna, it is probable that almost any filarioid nematodes parasitizing animals can, under proper circumstances, infect humans and undergo some degree of development, and undoubtedly, additional species will continue to be isolated from humans in the future [38]. Consequently the dynamics of transmission, the roles of various definitive and intermediate hosts, immunological factors associated with infection, and possible pathways for anticipation and prevention of outbreaks should constitute the focus for future studies of these parasites and may contribute insights for modeling of human filariosis.

## Conclusions

*Rumenfilaria andersoni* along with *Setaria tundra* are prominent filarioid nematodes now recognized to be widespread in Finland among artiodactyle hosts. In addition to semi-domestic reindeer, lymphatic dwelling *R. andersoni* was identified for the first time in Europe among endemic moose, wild forest reindeer and roe deer and in the introduced population of white-tailed deer. The parasite appears to have been originally introduced and established in Finland coincidental with translocation of white-tailed deer from North America in 1935. Subsequent expansion, involving both geographic and host colonization, has to a large part been driven by accelerating climate warming. Shifts in temperature directly determine parasite development and opportunity for transmission mediated by interactions of swarming hematophagous insect vectors and ungulate definitive hosts in the context of increasingly permissive environments. A signature for south to north expansion in Finland is apparent with invasion and emergence over the past decade being related to both cumulative and extreme climate/weather events. Emergence of *R. andersoni* in reindeer occurred simultaneously with an outbreak of *S. tundra,* suggesting common factors of temperature and humidity although different vectors are indicated by the discrete circadian periodicity of microfilaremia associated with these filarioids.

We demonstrate that parasite life history is characterized by intense infections of adults and microfilariae in ungulate hosts, an extended prepatent period near 5 months, and considerable longevity for mature nematodes. A midsummer window for transmission is supported by the seasonally defined peak in rmf density observed in reindeer, and by the well-known attacks on herds of caribou/reindeer by massive swarms of blood-feeding insects. Among moose and white tailed deer the overall prevalence and intensity was substantially lower than that observed among reindeer. This suggests the nematode may be adapted to these Alceini and Odocoileini, and that these cervids may be among the primary hosts of *R. andersoni*; high intensity infections in reindeer may reflect a recent association with naïve hosts. The pathological impact of *R. andersoni* on reindeer health and well-being remains unknown, although it is highly probable that substantial density of rmf may have negative systemic effects, consistent with other filarioids in wildlife species and humans. Our study highlights the interactions of climate and host-parasite biology, providing a deeper understanding for processes of expansion and emergence and recognition of common invasion pathways in northern systems.
